# Development and Progression of Polymer Electrolytes for Batteries: Influence of Structure and Chemistry

**DOI:** 10.3390/polym13234127

**Published:** 2021-11-26

**Authors:** Gregory Rollo-Walker, Nino Malic, Xiaoen Wang, John Chiefari, Maria Forsyth

**Affiliations:** 1 Institute for Frontier Materials, Deakin University, 221 Burwood Highway, Burwood, VIC 3125, Australia; grollowalker@deakin.edu.au (G.R.-W.); xiaoen.wang@deakin.edu.au (X.W.); 2 CSIRO Manufacturing, Bag 10, Clayton South, VIC 3169, Australia; Nino.Malic@csiro.au (N.M.); John.Chiefari@csiro.au (J.C.)

**Keywords:** polymer electrolyte, block copolymers, lithium ion, battery, ionic polymer, solid-state battery

## Abstract

Polymer electrolytes continue to offer the opportunity for safer, high-performing next-generation battery technology. The benefits of a polymeric electrolyte system lie in its ease of processing and flexibility, while ion transport and mechanical strength have been highlighted for improvement. This report discusses how factors, specifically the chemistry and structure of the polymers, have driven the progression of these materials from the early days of PEO. The introduction of ionic polymers has led to advances in ionic conductivity while the use of block copolymers has also increased the mechanical properties and provided more flexibility in solid polymer electrolyte development. The combination of these two, ionic block copolymer materials, are still in their early stages but offer exciting possibilities for the future of this field.

## 1. Introduction

Battery technology is currently at a crossroads; there is a greater demand for these devices to have increased performance, particularly in overall capacity, but also a need to address safety concerns and life cycle sustainability [1]. The majority of current commercial Li-ion devices operate using a liquid solvent-based electrolyte system [2]. This technology has satisfied most applications, from batteries for personal electronics to electric vehicles and grid storage. However, as current societal demands point to an increasing need for renewable energy and decarbonization of the economy, a key component to meet this demand has been significant battery development for the electrification of transport [3] and backup grid storage using battery farms [4]. As the need for greater energy density and capacity continue to grow, safety concerns associated with current Li-ion technology becomes a more significant issue. One of the key stepping-stones in the road towards next-generation technology that addresses the need for improved performance and safety is the development and commercialization of solid-state batteries with non-flammable solid electrolytes [5,6].

Polymer electrolytes are unquestionably a primary candidate for these new technologies, with their ease of processing and low flammability [7]. On top of this, polymers offer structural versatility ranging from straight linear homopolymers to advanced architectural block copolymers allowing for fine-tuning of select properties [8]. Polymer electrolytes can generally be classified under three broad classes of electrolyte materials: solid polymer electrolytes (SPEs), materials solely made up of polymer and target salts (e.g., lithium/sodium salt); gel polymer electrolytes (GPEs), with additional liquid components; and composite polymer electrolytes (CPEs), with the incorporation of functional organic and inorganic fillers [9,10,11].

As with all solid-state alternatives, a major challenge lies in replicating the ion transport properties of the liquid state. This has been approached both through the trialling of various additives with traditional polyether-based systems [12] and the evolution of different polymer chemistry such as polycarbonates [13], ionic polymers [14,15], and block copolymers [16,17]. This review will look to show the development of these materials and demonstrate the benefits of various structures and chemistries as well as highlight their shortcomings.

## 2. Polymer Electrolytes

The foundation for this field of research began with the initial discovery of ionic transport and hence conductivity in a polymer by Fenton et al. in 1973 through the complexation of sodium and potassium salts in polyethylene oxide (PEO) [18]. This was followed by work from Armand et al. using lithium salts dissolved in PEO to prepare conductive electrolytes which were applied to produce the first prototype polymer battery [19]. At this point, polymer electrolytes were seriously considered as the next step of enabling solid-state battery technology, which led to significant materials research directed this way in the 1980s and early 1990s.

**Figure 1:**
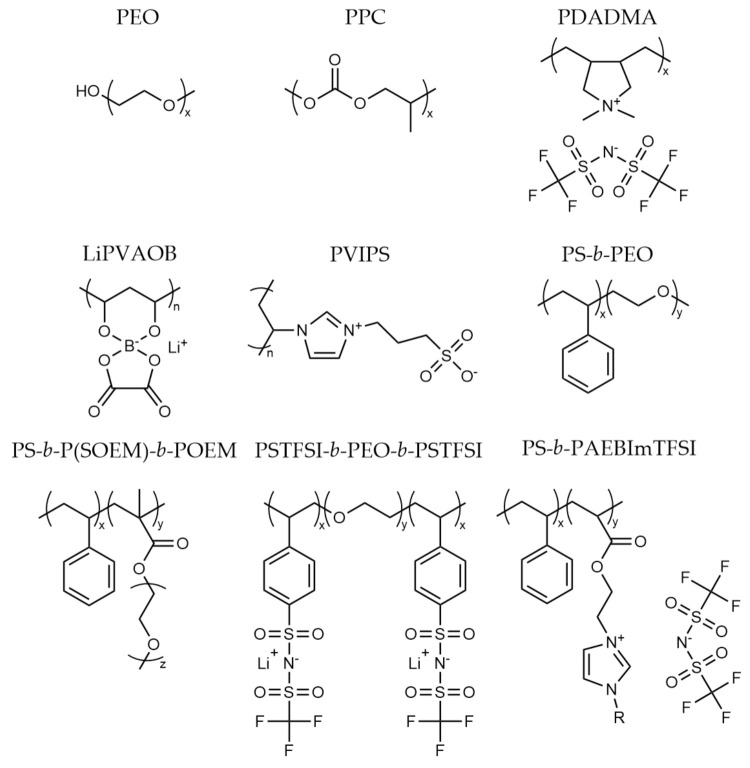
Chemical structures of the polymers described in Table 1. PEO: polyethylene oxide; PPC: poly (propylenecarbonate); PDADMA: (polydiallyldimethylammonium) bis (trifluoromethanesulfonyl)imde; LiPVAOB: lithium polyvinylalcohol oxylate borate; PVIPS: poly(vinylimidazolio) propanesulfonate; PS-*b*-PEO: polystyrene-*block*-polyethylene oxide; PS-*b*-P(SOEM)**-***b*-POEM: polystyrene-*block*-polystyrene-*grad*-poly(oligo-oxyethylenemethacrylate)-*block*-poly(oligo-oxyethylenemethacrylate); PSTFSI-*b*-PEO-*b*-PSTFSI: poly (4-styrenesulfonyl) (trifluoromethanesulfonyl imide)-*block*-polyethylene oxide-*block*-poly (4-styrenesulfonyl) (trifluoromethanesulfonyl imide); PS-*b*-PAEBImTFSI: polystyrene-*block*-poly (acrylethyl (butylimidazoliumbis (trifluoromethanesulfonyl) imide)).

The early development of these materials focused primarily on high-solvating polymers, typically PEO (Figure 1) and various lithium salts. While these materials reached reasonable conductivity (≈10^−4^ Scm^−1^) at elevated temperatures (60 °C), the values plummet at ambient temperature (≈10^−7^ Scm^−1^) due to polymer crystallinity [19]. Moreover, lithium transport numbers (i.e., the number of moles of lithium transferred by migration per Faraday of charge or the fraction of current carried by the lithium ions [13]) are relatively low (0.2–0.3), showing that the anion has a major contribution to the measured conductivity [20]. Use of complexing additives such as glymes and carbonates have been introduced into these systems to reduce the interaction between Li-ion and the oxygen atoms on the PEO backbone, allowing for higher Li-ion mobility [21,22]. Alumina and other inorganic fillers added to PEO-based electrolytes have also been shown to improve ionic conductivity by an order of magnitude through the formation of localized amorphous regions reducing the PEO crystallinity [23,24]. Work has also progressed towards altering the polymer architecture and through the introduction of copolymers. Alternative polymers that have been investigated include polycarbonates [25,26] and polynitriles [27,28], among others. More recently, interest in ionic polymers based on polymerized ionic liquids has developed due to their high ionic conductivity when combined with other components including lithium salts and ionic liquids [29,30,31].

As these polymeric materials have been developed over time, they have demonstrated favourable properties such as low flammability, ease of processing, and electrochemical stability [7,32]. They also offer greater mechanical tolerance to electrode deformation than liquid alternatives [33]. In addition, their general flexibility offers greater interfacial contact with either electrode compared to other solid-state counterparts [34]. There remain key areas for improvement in these materials such as increasing the effective Li-ion conductivity (i.e., increased Li-ion transport number) to overcome polarization due to anion migration and the mechanical strength to suppress lithium metal dendrites, two of the biggest challenges [35].

Polymer electrolytes have extended beyond lithium-based devices with recent reports highlighting their use in sodium metal batteries [36,37]. Although sodium batteries offer a lower capacity, they can be much more sustainable due to the greater abundance of sodium. Initial results are promising, with reports of stable solid electrolyte interphase (SEI) and high-capacity retention [38,39]. As with the Li-ion counterparts, improvements in ion mobility and mechanical strength are required, but the area is still relatively unexplored [37].

The polymer electrolytes used in battery materials so far (summaries of example chemistries and structures are shown in Table 1 and Figure 1) have been focused on a relatively narrow group of polymers while there is still a plethora of chemistries and structures to explore. The remainder of this review will discuss the more promising polymer materials and chemistries that have been investigated as well as outline the emerging materials for next-generation polymer electrolytes.

### 2.1. Polyethylene Oxides—PEOs

**Figure 2:**
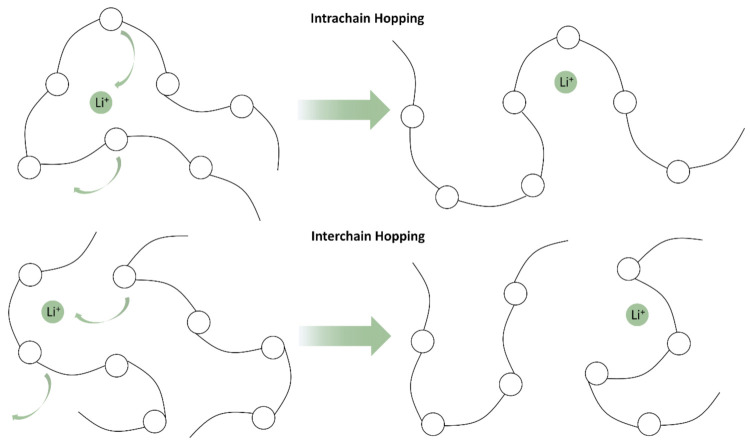
Mechanisms for ion transport in PEO.

Since its initial discovery, PEO and its derivatives have been commonly put forward as the foundation material to build a solid polymer electrolyte [18,19]. This is primarily based on its strong solvating properties for Li-ion but is backed up by high chain flexibility that promotes ion transport. In a PEO system, Li-ions are coordinated by the ether-group oxygen atoms on the polymer backbone and are transported through both an inter- and intra-chain hopping motion (Figure 2) [49]. While other oxide polymers, such as polymethylene oxide (PMO) and polypropylene oxide (PPO), have the same ether oxygen to coordinate to the Li-ion, the helical wrapping achieved by PEO due to the optimal spacing of two carbons between each oxygen maximizes the solvation properties [50].

Ion transport is the fundamental property of an electrolyte and, as such, increasing the ion transport of these systems has always been paramount. However, even when a polymer material has a very high ionic conductivity, if the transport number of the target cation is low, then the material will be a poor battery electrolyte [51]. This transport number can be defined as the mobility of the target ions relative to the other ions in the material [52]. As highlighted earlier, lithium transport numbers in these systems are generally low, at 0.2–0.3, which points to the anionic component of the lithium salt playing a significant role in the overall conductivity of the polymer electrolyte. Whilst traditional lithium salts such as LiPF_6_ have been considered [36], perfluoro-alkyl sulfonyl imide salts Li(N(SO_2_C_n_F_2n+1_)_2_ are more commonly employed with polymer materials, as these larger anions can better dissociate in the polymer matrix. Furthermore, these salts cause disruption in the crystallinity, lowering the melting point, *T_m_*, in PEO-based electrolytes, resulting in higher ionic conductivity at lower temperatures while demonstrating good thermal and electrochemical stability [53].

For these simple binary polymer electrolyte systems, the transport mechanism follows a diffusive process through the segmental motion of polymer chains. Thus, crystallinity in a polymer electrolyte limits ion dynamics, and materials with a higher amorphous fraction display a higher ionic conductivity [13]. As a result, phase transitions such as the *T_g_* and *T_m_* have a great impact on ionic conductivity; for instance, a lower *T_g_* leads to increased dynamics at ambient temperatures and hence higher conductivity. Given that ion dynamics are coupled to polymer segmental motion and *T_g_*, conductivity typically follows Vogel-Fulcher-Tamman (VFT) behaviour, as described by Equation (1) [54].

(1) $$\sigma =\sigma_{0}exp\left(\frac{-B}{T-T_{0}}\right)$$

The ideal ionic conductivity of such materials is to match that of liquid electrolytes at room temperature (≈10^−3^ − 10^−4^ Scm^−1^) [9]. For primary PEO–Lithium salt systems, this objective is only achieved at elevated temperatures (>60 °C) around and above the *T_m_*. However, at this point, the mechanical strength of these films is compromised, meaning applications are limited. This has led to alternative strategies to improve conductivity through lowering the *T_g_*, using additives such as plasticisers, suppressing crystallinity, and increasing mechanical properties by the addition of inorganic fillers and designing polymer blends and composites [55].

The role of a plasticiser is to soften and increase the flexibility of the polymer by lowering the *T_g_*_._ This in turn should increase ion mobility through the polymers [56]. Typically, plasticisers are small molecule species that have secondary interactions with the polymer chains, leading to increased mobility [57]. Low molecular weight PEO (also referred to as PEG) has successfully been used as well as other aprotic organic solvents such as ethylene carbonate (EC) and propylene carbonate (PC) to achieve improved conductivity at ambient temperatures (Table 2) [58,59]. However, these additives typically reduce the mechanical stability of the materials and although carbonates are not as flammable as other organic solvents, they still have a low flash point and are corrosive, thereby increasing the risk of possible battery fire and damage [60]. Ionic liquids (molten salts at room temperature) have also been targeted as plasticisers due to their high ionic conductivity and negligible volatility [61,62]. Their combined chemical and thermal stability make them an appealing option concerning safety [63].

Other polymers have also been blended with PEO to disrupt the inter-chain interactions and increase their amorphous phase. Early examples included materials such as poly (methacrylic acid) (PMAA), which introduced hydrogen-bonding interactions that reduced PEO crystallinity [64]. Other approaches, including blending with poly (vinylidene fluoride) (PVDF), which appears to have good compatibility with PEO as well as wide electrochemical stability, have been well summarized in a recent review [60]. These blends have some of the highest ionic conductivities, especially with the LiTFSI salt [65,66]; however, the interaction between the Li and backbone fluorine atoms as well as its inherent crystallinity results in reduced Li-ion mobility [67]. The use of cellulose has also been used as a mechanical stabiliser in order to promote cyclability, although it has not offered significant gains in ionic conductivity [68]. Elsewhere, nanostructured polymers of siloxanes [69,70] and phosphazenes [71] have been trialled, offering minor improvements in ion transport.

Inorganic additives, such as fillers and matrices, which form composite polymer electrolytes have also led to good performance, generally improving mechanical properties while enhancing ionic conductivity by introducing further conduction pathways [72]. Metal oxides have been used most commonly with alumina (Al_2_O_3_) and titania (TiO_2_) systems, increasing ion conductivity by 1–2 orders of magnitude whilst also improving the elastic modulus [40,73]. Metal–organic frameworks (MOFs) have also been investigated but do not show as significant an improvement as metal oxide systems [74].

### 2.2. Polycarbonates

**Figure 3:**
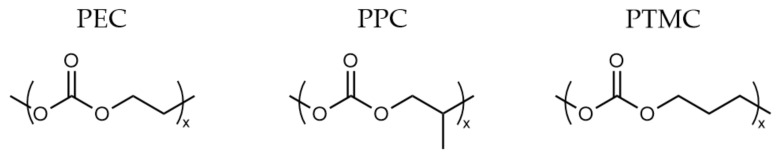
Different types of polycarbonates used as polymer electrolytes (PEC: polyethylene carbonate; PPC: poly (propylene carbonate); PTMC: poly (trimethylene carbonate).

Research into various polycarbonate-based solid polymer electrolytes (Figure 3) has significantly increased over the last decade as an alternative to PEO [13]. In a similar fashion, the Li-ion coordinates with oxygen on the polymer, preferentially to the oxygen in the carbonyl functional group. The extent of Li-ion coordination and hence the strength of the interaction can be identified and measured through vibrational spectroscopy [75]. This allows for an easy way of undertaking structure–property analysis for these materials to improve Li-ion transport. While the conductivity of neat polycarbonate electrolytes is lower than polyethers, their advantage is higher electrochemical stability and lithium transport numbers (>0.5). This is theorized to be due to loss of chelation and weaker interaction between the Li-ion and oxygen atoms in PC electrolytes [13].

**Figure 4:**
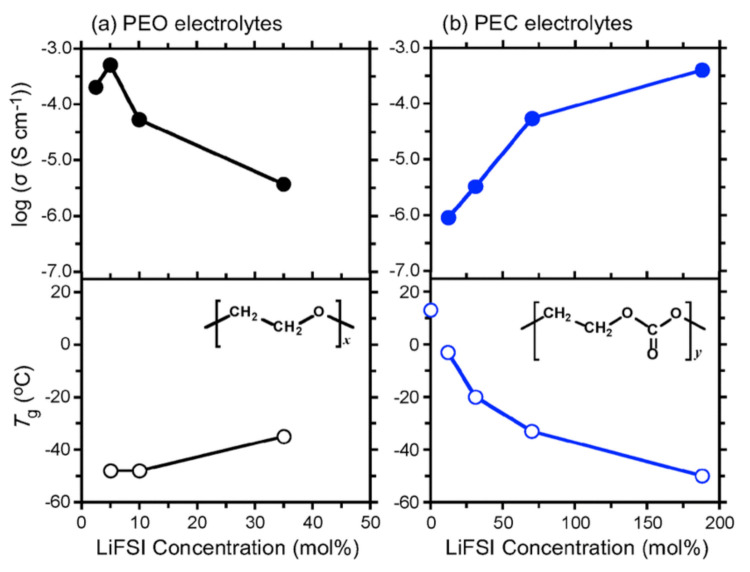
Dependence of ionic conductivity (at 60 °C) and *T_g_* on concentration of added LiFSI in (**a**) PEO and (**b**) PEC electrolytes. Reproduced with permission from Ref. [76]. Copyright 2014 Royal Society of Chemistry.

Polyethylene carbonate (PEC, Figure 3) and its derivatives have been a focus of research due to the unusual properties exhibited by them upon the addition of select lithium salts. Typically, polyether materials stiffen, reducing ion mobility upon the high loading of lithium salts. However the opposite is true in the addition of LiBF_4_, LiFSI, and LiTFSI to PECs, decreasing the *T_g_* and raising ionic conductivity, demonstrating plasticising behaviour (Figure 4) [76]. As such, PECs can have a greater salt loading, which in turn raises the conductivity of these systems, reaching working levels of 2.2 × 10^−4^ Scm^−1^ at 60 °C for a PEC_0.53_LiFSI system [77].

The use of a cellulose matrix with poly (propylene carbonate) (PPC, Figure 3) by Zhang et al. demonstrated promising conductivity (3.0 × 10^−4^ Scm^−1^) at room temperature, despite having a high *T_g_* (5 °C) for a polymer electrolyte material [41]. However, other work with PPC materials without a cellulose matrix has not yielded such impressive results. Thus, the enhancements are most likely being provided by other additives or structural changes and not the polymer [13].

Poly (trimethylene carbonate) (PTMC, Figure 3) is another main chain polycarbonate material that has been investigated. It offers more varied polymerization techniques due to the ring strain associated with its monomers and is predominantly amorphous with a *T_g_* below zero [78,79]. These materials also have a much greater oxidative stability than ether-based polymers, with oxidation only occurring above 4.5 V [79]. However, the ionic conductivities of such materials are generally lower than PEC due to the lack of the same plasticising effect upon the addition of Li salt. Nevertheless, excellent transference numbers have demonstrated ample lithium transport kinetics for full-cell cycling (Li/LiFePO_4_) [80,81]. Furthermore, PTMC-based electrolytes have also been implemented in sodium metal batteries successfully and cycled with reasonable capacity retention (>80%) [82].

While the drive for initial polymer electrolytes and PEO focused on salt solvation, polycarbonates demonstrate that good electrolyte performance requires more than just that. Indeed, a lower solvation strength between the Li-ion and polymer allows for greater target ion transport in these systems.

### 2.3. Ionic Polymers

**Figure 5:**
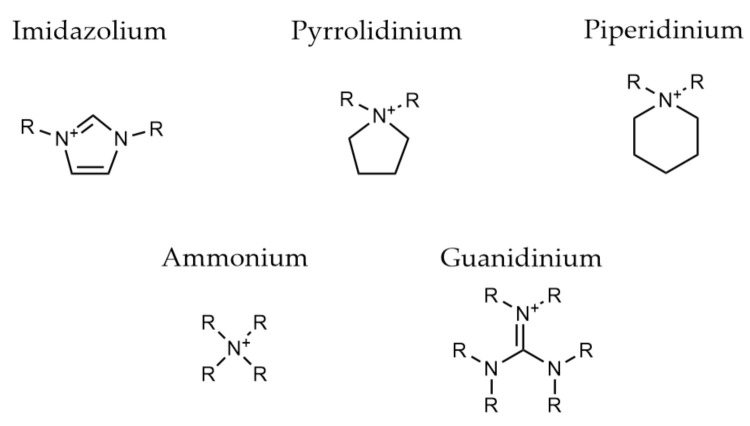
Typical ionic liquid cation bases that have been translated to ionic polymer electrolytes.

One of the driving forces behind the use of polymer electrolytes is the benefits of enhanced safety. As shown already in this review, it has been difficult to achieve ionic conductivity levels close to liquid electrolytes without further additives. However, the incorporation of ionic components into a polymer, e.g., as shown in Figure 5, provides ionic polymers (IPs) with a combination of good ionic conductivity alongside the primary properties a polymer material offers. When these materials are polymerized from ionic liquid monomers, they are often referred to as polymeric ionic liquids or poly (ionic liquids) [83]. Other types of ionic polymers can be produced from polymeric precursors via a post-polymerization functionalisation. Their unique properties have led to increased research over the last decade, in particular in their use as battery electrolyte materials [84,85].

**Figure 6:**
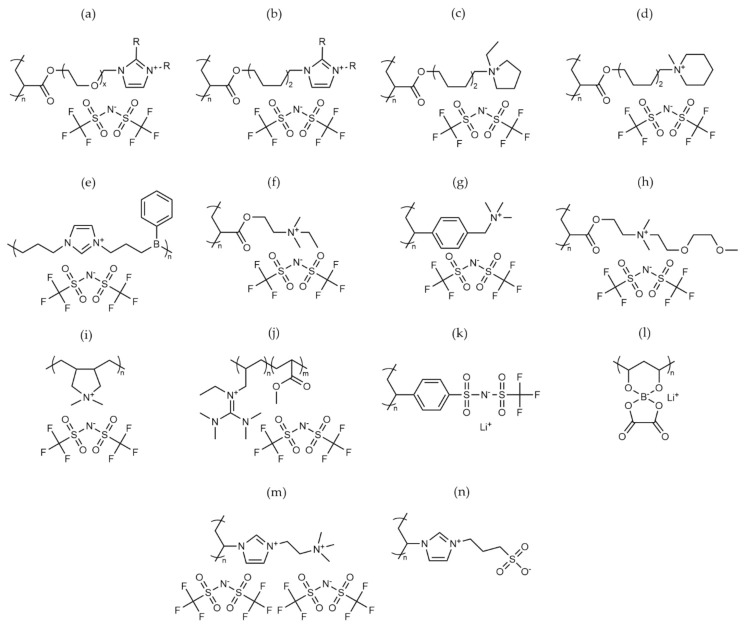
Chemical structures of selected ionic polymers (**a**–**n**) that have been analysed as solid polymer electrolytes in this review.

Ohno et al. pioneered the early work on these polymers looking at nitrogen-centred cation species [86,87,88,89,90]. They determined that through the extension of the cation-polymer backbone spacer, ionic mobility could be increased due to rotational freedom of the cation ‘brush’ (see Figure 6a [89]). In terms of the chemistry of the ionic polymers, those with an imidazolium-based cation demonstrated a higher ionic conductivity when compared to piperidinium and pyrrolidinium (Figure 6b–d) analogues [86]. This trend was also observed for electrolyte systems with LiTFSI salt; however, piperidinium cation systems offered a higher lithium transport number (0.43 vs. 0.11 at 25 °C), balancing out any loss in conductivity through lithium mobility. The mechanism for this behaviour was unclear. Further work to increase the transport number of these materials involved the introduction of a Lewis acid to act as an anion trapper. A number of alkylborane materials were synthesised using hydroboration polymerization with the use of a mesitylborane side group (Figure 6e), demonstrating a very high lithium transport number (0.87 at 30 °C) [91].

As well as the ring-centred nitrogen ionic species, tetraalkylammonium analogues have also been tested. Zhang et al. synthesised an acrylate-based ammonium IP (Figure 6f) which, when paired with an ammonium IL (N_1222O1_TFSI), offered appreciable conductivity (10^−5^ Scm^−1^ at 25 °C) while it was stable up to 4.0 V (vs. Li^+^/Li^0^); it also showed in a Li/LFP full cell promising cycling performance, at an elevated temperature (60 °C), with over 120 cycles and an average discharge of ~125 mA hg^−1^ and 99% efficiency at 0.1 C [92]. Similar electrolyte performance has also been observed for an analogous species, whereby the acrylate was swapped for a polystyrene alternative (Figure 6g), suggesting that IP performance is not impacted by the backbone component of the polymer [93]. However, the initial work carried out no mechanical analysis on the polymer, so no conclusions can be drawn to the effect of the backbone on the structural integrity of the systems. Work on similar systems from Nie et al. suggests that the addition of ether functional groups (Figure 6h) onto the ionic brush can lead to stable high conductivity with a lower *T_g_* and good stability against lithium metal when LiFSI or LiTFSI salt was paired with the IP [94]. This binary polymer/salt composition also achieved a higher oxidative stability 4.7 V vs. Li^+^/Li^0^ with LiFSI (5.2 V LiTFSI); however, this could be due to the stability of the ammonium IL additive, rather than a property intrinsic to the polymer.

Poly (diallyldimethylammonium)-based (PDADMA, Figure 6i) electrolyte materials were initially investigated in composites with tetraglyme and offered good potential [95]. Further work into solid-state devices has been performed by Mecerreyes et al. [96]. These materials were synthesized via a chloride precursor that was commercially used in water purification processes and could be converted into a potential electrolyte material through anion exchange of the chloride with non-soluble alternatives. Through the addition of lithium salt (LiTFSI) to the polymer and further doping with a pyrrolidinium-based IL (C_4_mpyrTFSI), an ionic conductivity of 10^−4^ Scm^−1^ (20 °C) was achieved at 60%wt loading of the IL [97]. The electrochemical stability of these materials also demonstrates the potential for high-voltage applications with the system stable up to 5 V vs. Li^+^/Li^0^. Li metal plating and stripping experiments showed that the membrane is capable of withstanding long-term cycling (>2000 cycles) without the formation of dendritic species, albeit at low-current densities. Good capacity retention was achieved when full-cell cycling was carried out in a Li/LiFePO_4_ all solid-state device at 40 °C; however, there was a significant loss in nominal capacity above a charge/discharge rate of C/5, suggesting that the material limiting current density is just above this (0.176 mA cm^−2^) [97]. Further work on this material has incorporated a small amount of Al_2_O_3_ to improve mechanical properties while maintaining the lithium transport number [98]. An electro-spun mechanical PVDF framework for the PDADMA TFSI polymer (Figure 6f) has also been employed to improve the mechanical properties of the material. This in turn allowed for greater concentrations of lithium salt and, as such, an improved Li-ion transport number, leading to higher Li-ion conductivities [42].

Ionic polymer derivatives of guanidinium ILs (Figure 6j) have been explored through copolymerization of ionic monomers with methyl acrylate by Li et al. [99,100]. Initial work from this showed promising conductivity when paired with a LiBF_4_ salt with a conductivity of 10^−4^ Scm^−1^ at 30 °C. The oxidative stability of the polymer was shown to be 4 V vs. Li^+^/Li^0^, which was independent of the counter-anion [99]. Further cycling performance at 80 °C using a TFSI-based system with an LFP cathode showed relatively stable capacity retention at C/10; however, this dropped off quickly at charging rates greater than C/10 [100].

Aside from nitrogen cations, the use of anionic species in ionic polymer electrolytes is of significant interest as these polymers can act as single-ion conductors. As such, when the anionic backbone is paired with a lithium cation and without further addition of salts or ionic plasticisers, the sole source of ionic transport is from lithium mobility. This means that the lithium transport number of these materials will approach unity (t_Li_ = 1), thus removing the issue of anion polarization (build-up of ion concentration around an electrode), which is a common source of battery failure when the anion is more mobile [101]. One approach to achieve this type of material is through the polymerization of a styrenic monomer functionalised with a perfluoro-alkyl sulfonyl imide group that is analogous to a TFSI anion (Figure 6k). However, the ionic conductivity of such systems is very low, even when doped with PEO to form a polymer-blend material (10^−8^ Scm^−1^–25 °C) [102]. An alternative anion chemistry that has been explored is the use of borate grafted to a polyvinylalcohol (PVA) backbone (LiPVAOB , Figure 6l) [43]. As electrolyte salts such as borate anions have received particular interest for their favourable SEI formation in stabilizing graphitic anodes [103]. As a polymeric system, the LiPVAOB electrolyte demonstrated very high oxidative stability (7 V vs. Li^+^/Li^0^), with a conductivity of 10^−6^ Scm^−1^ at ambient temperatures. However, the thermal stability was significantly lower than other polymer systems, with degradation beginning at only 100 °C, although it should be noted this is still higher than current solvent-based systems [43].

Polymer electrolyte investigations have not been merely limited to those having a single type of ionic species on the polymer backbone; for example, those which have different poly-cation and –zwitterion polymer materials are also developed and tested. For example, di-cationic species on a polymer brush have been explored by Yang et al. using an imidazolium and tetraalkylammonium (Figure 6m) group as each ionic component. This material was reported to have a higher charge density and increased IL loading ability versus a single cation imidazolium polymer. As a result of the IL loading, strong interface properties were achieved and full-cell cycling Li/LFP at room temperature was carried out with a near theoretical discharge capacity of 161 mA hg^−1^ at the 50th discharge [104]. In addition, the use of zwitterionic (cationic and anionic functionality covalently bonded with each monomeric unit) polymers has been investigated. On a molecular level, zwitterions have been shown to reduce non-target ion mobility, ensuring single-ion conduction as well as reducing SEI layer resistance [105]. By tethering these into a polymerized system (Figure 6n), these materials offered good ion conductivity at 30 °C, although they suffered from significant capacity fade when incorporated into a Li/LFP full cell [44].

### 2.4. Block Copolymers

A trend of seeking high ionic conductivity and lithium transport is observed throughout the various types of homopolymers explored thus far in this review. However, the long-standing goal for solid-state technology and polymer electrolytes is to be used with lithium metal serving as the anode to achieve a safe high energy density rechargeable battery. While sufficient ion transport is required, the dendrite formation observed with lithium metal batteries is the prime reason for cycling failure. This fault is caused by the non-uniform plating and stripping of lithium metal. Kinetic modelling work by Monroe et al. has shown that dendrite formation may be suppressed when the shear modulus of an electrolyte exceeds 7 GPa [106]. As already described, while current homopolymer electrolytes have achieved good conductivity, this has often been paired with insufficient mechanical strength without further reinforcing frameworks or additives.

**Figure 7:**
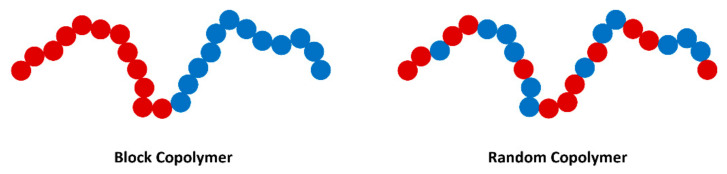
Schematic illustrating difference between block copolymers (BCP) and random copolymers.

The introduction of defined block copolymers (Figure 7) offers the advantage that a single macromolecule (i.e., not blended) will have properties that multiply a combination of the block components. However, for a random or statistical copolymer (Figure 7), the macromolecule does not display distinct properties, as in the case of a block copolymer [107]. This structural difference has significant implications on the suitability of these materials as ion-conducting solid polymer electrolytes, as is evidenced by being investigated as an electrolyte with the desired mechanical and electrochemical properties [45]. These materials are typically comprised of two opposite phases: a ‘soft’ block containing sequential polar functional groups for salt solvation and conduction pathways and a ‘hard’ block to provide elastic strength. Early work on BCPs as electrolytes can be seen as far back as the 1980s with polystyrene (PS) used in ABA triblock materials with ether grafted polybutadiene and poly (oligo (oxyethylene)methacrylate). However, at this early stage, conductivity was the primary testing factor, with no apparent work on mechanical analysis [108,109].

**Figure 8:**
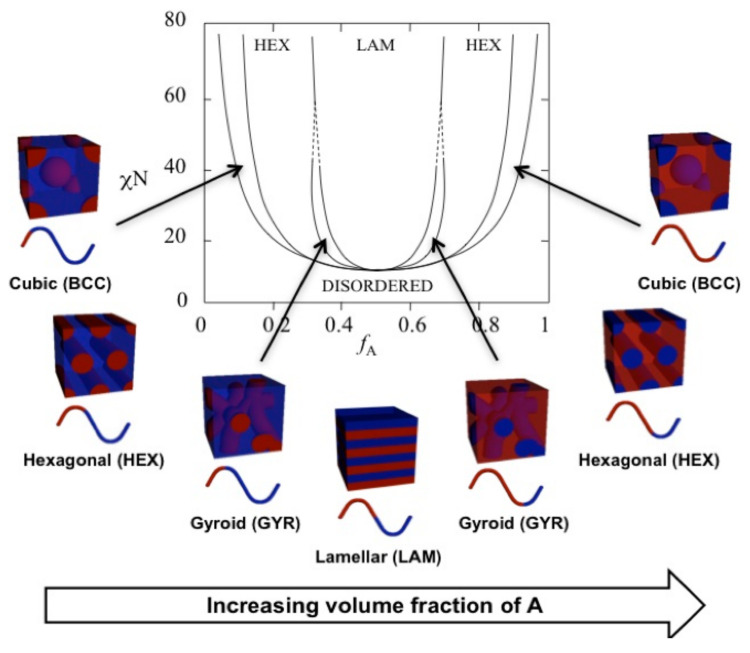
Schmatic demonstrating the effects of block interactions and the volume fraction of each phase on the nanoscale network structure. Reproduced from Ref. [112].

When the chemical properties of each polymer block differ significantly, there is a degree of self-assembly of the material, allowing for the potential of structural design to tune ion transport and mechanical characteristics. The morphology of these BCPs is dictated by the following parameters: Flory–Huggins interaction parameter (χ), the degree of polymerization of each block, the volume fraction of each block, and the specific architecture [110,111]. Depending on the composition, the morphology can adopt a range of ordered systems, as shown in Figure 8, as well as a disordered state. Therefore, through careful synthetic control of a BCP, it is possible to maximise certain characteristics through the nano-scale morphology.

The use of BCPs over random or statistical copolymer structures has a vast impact on the conductivity of the materials (Figure 8). Work by Choi et al. [113] investigated a BCP and its random copolymer counterpart and demonstrated that the BCP network morphology resulted in an increase in conductivity by around two orders of magnitude. Overall, the ion conduction pathways are made more complicated in BCPs by the introduction of non-conductive block segments in the electrolyte matrix. These transport systems can be split into two routes: intra-grain, where ions are carried along the conductive pathways. and inter-grain, the path across these conductive sections [16]. For intra-grain, two-dimensional lamellar phases have traditionally been viewed as highly conductive since they offer a continuous diffusion pathway. However, this requires the correct alignment of nanoscale structure over multiple grain boundaries to ensure conduction, which is a significant challenge to extend onto the larger scale required in commercial battery device production. A solution is the use of three-dimensional domains, which rules out the need for precise orientation. A double-gyroid morphology is the perfect example of this and has been shown to have the highest diffusion coefficients if the volume fraction of the conducting block is maximized [114,115].

In recent times, the synthesis of BCPs has been greatly enhanced through the use of ‘living’/controlled polymerization techniques. The “living” nature of this polymerization results in greater precision over each block length, allowing for greater control over volume fraction than traditional polymerization techniques [116]. These controlled methods have been implemented across anionic [117], cationic [118] and radical [119] polymerization techniques. Of these, the free radical polymerization technique, known as reversible addition–fragmentation chain transfer (RAFT), has been widely implemented as a result of its versatility in obtaining a range of BCP compositions and structures [120].

The monomer of choice for the hard block component has typically been styrene and its derivatives for many BCP electrolytes due to its high elastic modulus up to temperatures of 100 °C. While the combination of different blocks with polystyrene reduces the mechanical properties compared to pure polystyrene, overall, these materials are far more robust than current homopolymer electrolytes, displaying elastic moduli approaching 10^9^ Pa [106]. Alongside styrene, PEO has been predominantly used as the conductive block [16]. An early example of this was demonstrated by Wang et al. [121] in 2003, using a PS–*block*-PS(EO)–*block*-PS block-graft copolymer showing good mechanical properties (E’ = 10^8^ Pa) while offering modest ambient temperature conductivity (10^−5^ Scm^−1^).

**Figure 9:**
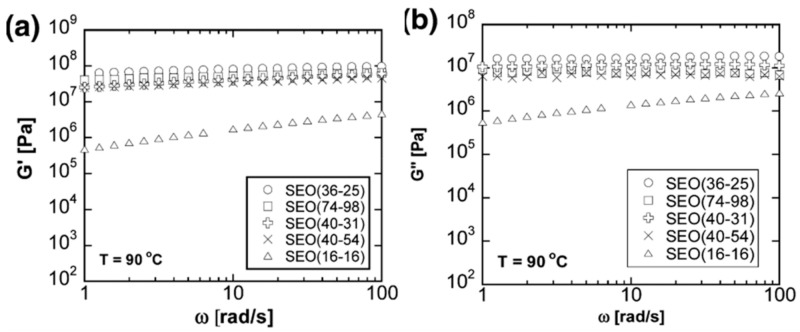
Effect of molecular weight on the (**a**) storage and (**b**) loss modulus on polystyrene–polyethylene oxide (SEO) block copolymer. Numbers in brackets relate to molecular weight in kgmol^−1^ of PS and PEO blocks respectively. Adapted from [45].

Further work with these combinations of monomers has explored the effects of molecular weight of PEO (M_PEO_) on the system [45,122]. These showed a positive correlation between M_PEO_ and ionic conductivity, although this trend tends to plateau at values of M_PEO_ > 60,000 gmol^−1^_._ While these works focused on the impact of PEO molecular weight, the mechanical analysis performed demonstrated the impact of polystyrene molecular weight as well (Figure 9). For most of these materials, the elastic modulus remained constant, other than when the styrene chain molecular weight was around 16,000 gmol^−1^, where there is a significant loss in the storage modulus. This is likely due to the molecular weight of the PS block being below its entanglement molecular weight, at which point the mechanical properties of polystyrene are reduced [123].

Additives such as ionic liquids have been used in BCPs as with homopolymers to improve the ionic conductivity. These materials have demonstrated selective solvation into the ‘soft’ block domains. This in turn has an impact on the volume fraction of each domain and hence the morphology [124]. Simone et al. [125] further explored this by combining various proportions of a PS–PEO BCP with 1-ethyl-3-methylimidazolium bis (trifluoromethanesulfonyl)imide (EMI–TFSI) and demonstrated that the lamellar and hexagonal nanostructures could be tuned accordingly.

**Figure 10:**
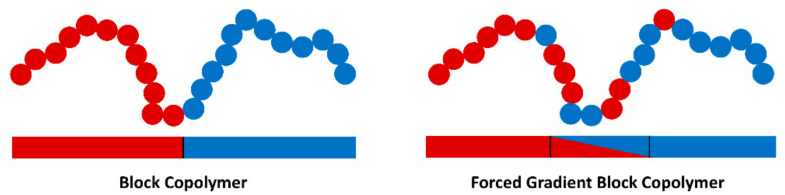
Scheme illustrating the difference between block copolymer and the forced gradient block copolymer produced by Epps et al. [46].

Outside the traditional block copolymer domain, Epps et al. [46,126] used copolymerization techniques to produce defined gradient sections in a range of BCPs (Figure 10). These materials have demonstrated tunable thermal and morphological properties through the control of the size and composition of this gradient section. This control over properties could be largely beneficial with regards to polymer electrolytes in order to optimise electrolyte performance [46]. In addition, the presence of a gradient section was observed to lower the *T_g_* and increase ionic conductivity compared to the neat block copolymer (Figure 10). The micro-phase separation between the two homopolymer block components was also shown to be present even when the volume fraction of the gradient block was over half of the total polymer (0.62).

### 2.5. Ionic Block Copolymers

**Figure 11:**
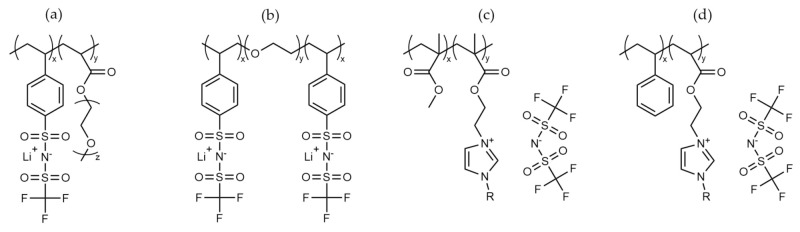
Chemical structures of different ionic block copolymers (**a**–**d**) materials that have been analysed as electrolytes in this review.

In a similar way, with developments in homopolymer electrolytes, ionic BCP alternatives have also been investigated, whereby either an ionic monomer has been used or an ionic component has been grafted onto the chain after polymerization (Figure 11). While some of these follow the principle of standard BCP electrolytes containing ‘hard’ and ‘soft’ blocks, others have sought to improve Li transport properties of more traditional homopolymers. The selection of tethered anionic species on the polymer backbone has been further investigated to produce ‘single-ion’ conduction materials by minimising the anions mobility [127,128].

Examples of such single-ion conduction materials have been successfully produced by grafting anionic moieties to the polymer backbone. As a result, the mobility of the anion is negligible; thus, any ionic conductivity is a result of the lithium transport alone. Feng et al. [102] successfully synthesised a copolymer material of [PSTFSI-*co*-MPEGA] (Figure 11a) which displayed near-unity Li transport. However, the ionic conductivity had a maximum of 7.6 × 10^−6^ Scm^−1^ at 25 °C, albeit without any extra salt or plasticisers. Meanwhile, Bouchet et al. [47] synthesised a BAB triblock copolymer [PSTFSI-*b*-PEO-*b*-PSTFSI] single-ion polymer electrolyte (Figure 11b). This too exhibited high lithium transport, and while a conductivity ≈10^−5^ Scm^−1^ was achieved at elevated temperatures, this quickly decreased at temperatures below 50 °C. The electrochemical stability of this polymer was excellent, with a voltage stability window up to 5 V with a storage modulus of 9 × 10^6^ Pa. This value is around an order of magnitude down from the non-ionic [PS-*b*-PEO-*b*-PS] discussed earlier in this review [45], suggesting that the ionic graft reduces polystyrene mechanical properties.

Work from Elabd et al. [113] has shown that self-assembly and microphase separation can be achieved for similar acrylate blocks through the introduction of an ionic functional group in one block [PMMA-*b*-PMAEBIm-TFSI] (Figure 11c). This material has been further developed by elongating the bridge between the polymer backbone and imidazolium groups with an extended alkyl chain. This has resulted in outstanding room-temperature ionic conductivity (1 × 10^−3^ Scm^−1^) for a solid polymer electrolyte, although the mechanical properties were not reported [129].

Additional BCPs featuring a hard block (PS) and ionic block (PMAEBIm-TFSI) (Figure 11d) have also been synthesised and characterised by Elabd et al. [113]. Further work on the electrolyte performance of this BCP system has been investigated by Forsyth et al. [48]. These polymers, with the addition of Li salt and IL, have offered a promising ionic conductivity of 10^−5^ Scm^−1^ and lithium transport > 0.5 at 50 °C. They have been further characterised through symmetrical and full-cell cycling, offering stable cycling and near-theoretical discharge capacity. The high calculated storage modulus of these polymers ≈3 × 10^6^ Pa was thought to contribute to the stability versus the lithium anode. Thus, the inhibition of the dendritic growth in these materials is likely a combination of the mechanical properties of the polymer alongside a sufficient internal pressure between the polymer electrolyte and lithium metal [130].

**Figure 12:**
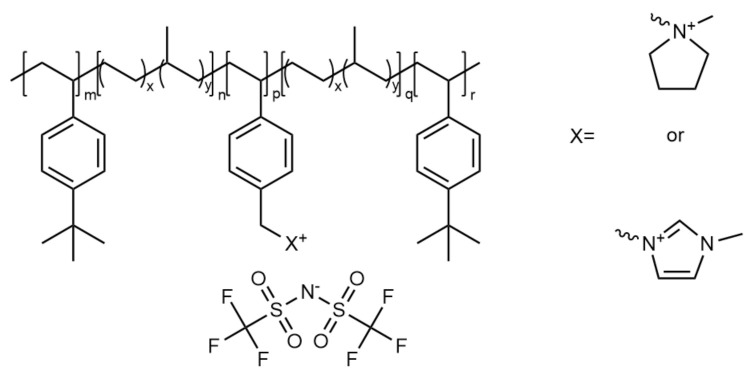
Chemical structure of ionic pentablock copolymer Poly(tbS-EP-mS-EP-tbS) electrolytes [131].

Elsewhere, a pentablock ABCBA ionic BCP (Figure 12) has been investigated to determine the effects of introducing further blocks on overall properties [131]. This too offered similar levels of ionic conductivity as other BCPs, and interestingly, with three polystyrenic units, the overall mechanical properties were not significantly improved. Therefore, the scope for multi-block copolymer systems beyond a triblock copolymer may not be necessary for improving properties and would avoid the additional production costs.

## 3. Summary

There has been a significant level of research directed at improving the ionic conductivity and mechanical stability of solid polymer electrolytes over the past three decades. The use of PEO and its derivatives as polymer electrolytes has been heavily investigated but ultimately, issues regarding the temperature range in which they operate and their electrochemical stability window remain. The introduction of polycarbonates helped improve the operational electrochemical window and lithium transport number, although this was paired with a loss in ionic conductivity. The field of ionic polymers is still growing and has shown strong ion transport but lacks mechanical robustness alone. Although research into a range of polymer classes continues, it is our belief that block copolymer systems offer the most viable option for an overall material that satisfies the general requirements for an effective solid-state electrolyte. The advent of controlled polymerization techniques such as RAFT offers an efficient and versatile method to make block copolymers with varying compositions and structures. Ultimately, this may offer the best way to optimise the important properties needed for a solid polymer electrolyte, which is mechanical strength and high Li-ion conductivity in the one polymer material. The development of ionic block copolymers is still in its infancy. Investigations into these materials continue to focus on structure–property relationships to ultimately develop a solid polymer electrolyte as a suitable membrane for lithium metal devices.

## Figures and Tables

**Table 1:** Comparison of some of the polymer electrolytes analysed in this review.

Polymer	Salt	Additive	*T_g_* (°C)	Ionic Conductivity (Scm^−1^)	Storage Modulus (Pa)	Reference
PEO	LiClO_4_	Al_2_O_3_	-	10^−5^ (30 °C)	-	[40]
PPC	LiTFSI	Cellulose	5	3.4 × 10^−4^ (25 °C)	2.5 × 10^7^ (25 °C)	[41]
PDADMA TFSI	LiFSI	C_3_mpyrFSI PVDF	−50	4.5 × 10^−4^ (25 °C)	10^5^ (25 °C)	[42]
LiPVAOB	-	PC	100	6.1 × 10^−6^ (25 °C)	-	[43]
PVIPS	LiTFSI	-		1.1 × 10^−4^ (50 °C)	-	[44]
PS-*b*-PEO	LiTFSI	-	-	3.6 × 10^−4^ (90 °C)	5.7 × 10^7^ (90 °C)	[45]
PS-*b*-P(SOEM)-*b-*POEM	LiTFSI	-	−54	10^−5^ (50 °C)	-	[46]
PSTFSI-*b*-PEO-*b*-PSTFSI	-	-		1.3 × 10^−5^ (60 °C)	9 × 10^6^ (40 °C)	[47]
PS-*b*-PAEBImTFSI	LiFSI	C_3_mpyrFSI	−26	7.6 × 10^−6^ (50 °C)	1.4 × 10^6^ (50 °C)	[48]

**Table 2:** Ionic conductivity of PEO–Lithium triflate electrolyte materials with various plasticisers.

Plasticizer	Ionic Conductivity (Scm^−1^)	Temperature (°C)	Reference
-	2.0 × 10^−5^	25	[58]
PEG	1.0 × 10^−4^	40	[59]
EC	3.2 × 10^−4^	25	[58]
EC/PC	4.0 × 10^−4^	25	[58]

## Abbreviations

Summary of abbreviations used throughout this review:

**Full Name**	**Acronym**
Solid Polymer Electrolyte	SPE
Gel Polymer Electrolyte	GPC
Composite Polymer Electrolyte	CPE
Ionic Polymer	IP
Block Copolymer	BCP
Ionic Block Copolymer	IBCP
Reversible Addition–Fragmentation Chain Transfer	RAFT
Glass Transition Temperature	T_g_
Melt Temperature	T_m_
Lithium Iron Phosphate	LFP
Polyethylene Oxide	PEO
Polyethylene Glycol	PEG
Polymethylene Oxide	PMO
Polypropylene Oxide	PPO
Ethylene Carbonate	EC
Propylene Carbonate	PC
Polymethylmethacrylate	PMMA
Polyvinylidene fluoride	PVDF
Polyethylene Carbonate	PEC
Polypropylene Carbonate	PPC
Polytrimethylene Carbonate	PTMC
Polydiallyldmiethylammonium	PDADMA
Polyvinyl alcohol	PVA
Polystyrene	PS
Bis(trifluoromethanesulfonyl)imide	TFSI
Bis(fluorosulfonyl)imide	FSI
